# Onset and relapse prevention of bipolar disorders

**DOI:** 10.1192/j.eurpsy.2023.115

**Published:** 2023-07-19

**Authors:** E. Vieta

**Affiliations:** Medicine, University of Barcelona, Barcelona, Spain

## Abstract

**Abstract:**

Early detection and intervention are critical for improved outcomes in mental disorders, and this is particularly true for bipolar disorders. Understanding the risk factors involved in the onset of the disease and the subsequent relapses and recurrences may lead to better results as regards to functional outcomes, which are the most relevant for patients and their significant others. This presentation will review those factors and discuss which ones can be used as targets for early intervention. It has been argued that, most often, early intervention comes late, and it is therefore paramount to build on strategies aimed at effective detection of people at risk and situations that may lead to recurring episodes of illness. Treatments not only aimed at treating symptoms, but at improving illness trajectories are necessary, and pharmacological formulations and interventions improving treatment adherence are particularly relevant to avoid interruptions of effective therapies.

**Disclosure of Interest:**

E. Vieta Grant / Research support from: Boehringer-ingelheim, Compass, Janssen, Lundbeck, Novartis, Consultant of: AB-Biotics, AbbVie, Adamed, Angelini, Biogen, Boehringer-Ingelheim, Celon Pharma, Compass, Dainippon Sumitomo Pharma, Ethypharm, Ferrer, Gedeon Richter, GH Research, Glaxo-Smith Kline, Janssen, Lundbeck, Medincell, Merck, Novartis, Orion Corporation, Organon, Otsuka, Roche, Rovi, Sage, Sanofi-Aventis, Sunovion, Takeda, and Viatris

